# Interconnection Technologies for Flexible Electronics: Materials, Fabrications, and Applications

**DOI:** 10.3390/mi14061131

**Published:** 2023-05-27

**Authors:** Ratul Kumar Baruah, Hocheon Yoo, Eun Kwang Lee

**Affiliations:** 1 Department of Electronics and Communication Engineering, Tezpur University, Assam 784028, India; 2 Department of Electronic Engineering, Gachon University, Seongnam 13120, Republic of Korea; 3 Department of Chemical Engineering, Pukyong National University, Busan 48513, Republic of Korea

**Keywords:** metal routing, flexible devices, flexible metal, flexible textile, flexible circuits, plastic substrates

## Abstract

Flexible electronic devices require metal interconnects to facilitate the flow of electrical signals among the device components, ensuring its proper functionality. There are multiple factors to consider when designing metal interconnects for flexible electronics, including their conductivity, flexibility, reliability, and cost. This article provides an overview of recent endeavors to create flexible electronic devices through different metal interconnect approaches, with a focus on materials and structural aspects. Additionally, the article discusses emerging flexible applications, such as e-textiles and flexible batteries, as essential considerations.

## 1. Introduction

Flexible electronics have become increasingly popular in various industries, including consumer electronics, sensors, soft robotics, healthcare, electronic skin, stretchable transistors, and more. The flexible electronics market has been rapidly growing in recent years due to market demands, and it is predicted that by 2026, the market will be worth USD 44.8 billion [1]. Flex circuits are designed to work on non-planar surfaces, which requires a flexible substrate. Materials such as silicon that are rigid cannot be used. The substrate material for flex circuits must have mechanical properties such as stretchability and self-healing, and must also be lightweight, resistant to oxidation, and biocompatible. The conducting material must be stretchable and highly efficient in conducting electricity with no changes in resistance when loaded, to meet the requirements of practical applications. Traditional conducting wires such as copper do not provide optimal mechanical and electrical performance. To address this, new architectures for conducting wires such as wavy, serpentine, honeycomb, and pre-stressed materials have been developed to enhance mechanical and electrical performance [2,3,4]. Each structure has its own merits and demerits, fabrication challenges, and design tradeoffs in terms of electrode density, cost, performances, etc. Recently, 2D intrinsic materials are looked at critically, for a prospective candidate as a conductive material that bears excellent mechanical and electrical properties [5,6]. Interconnects in e-textiles and their integration impact the performance of textile flex circuits [7]. Because of the flexible nature of flex circuits, the batteries have to be flexible yet high performing, and that needs innovation because existing rigid Li-ion batteries have limitations [8].

Metal interconnects play a crucial role in the functioning of flexible electronic devices by allowing electrical signals to flow between different components. When designing metal interconnects for flexible electronics, several factors need to be considered, including conductivity, flexibility, adhesion, reliability, and cost-effectiveness, as follows:

(i) Conductivity: Metal interconnects must have high electrical conductivity to minimize resistance and ensure efficient signal transmission.
(ii) Flexibility: Since flexible electronics are designed to bend and conform to different shapes and surfaces, metal interconnects must be flexible and bendable without breaking.
(iii) Adhesion: To ensure stable and reliable performance even under repeated bending and stretching conditions, metal interconnects must have strong adhesion to the substrate.
(iv) Reliability: Metal interconnects must be reliable and capable of maintaining their electrical performance over time, even under conditions of repeated use and exposure to a variety of environmental factors.
(v) Cost: Because flexible electronics are often manufactured in high volumes for consumer and industrial applications, metal interconnects must be cost-effective and scalable for mass production.

There are several metal materials commonly used for metal interconnects in flexible electronic devices, including copper, silver, gold, and aluminum. Each material has unique properties suitable for a particular application, and material selection will depend on the specific needs of the device. In this context, this review discusses recent developments in flexible electronics, including metal interconnects, flexible batteries, and e-textile applications, with a focus on materials, structures, and flexible integration technologies.

## 2. Flexible Electronics

Flexible electronics also known as flex circuits have evolved as a new dimensional technology in the modern era because of their attractive advantages such as being lightweight, bendable, twisted, twistable, stretchable, healable, or adapted into unique shapes. The electronics are implemented in an elastomeric substrate with strong molecular interactions, mostly plastic such as polyamide, flex glass, metal foil, paper, etc. This allows the circuit or the system to be mounted in stretchable substrates comfortably, which was not possible with systems taking rigid substrates such as silicon. Additionally, flex circuits can be screen printed on polyester. Moreover, identical components for electronic assemblies for rigid printed circuit boards can be used in flex circuits as per applications [9,10,11,12]. Flex circuits concepts have a modest beginning with Ken Gileo’s in 1903, who demonstrated an electronic device with flat metal conductors installed on paraffin-coated paper [13]. In 1948, Cledo Brunetti and Roger W. Curtis published an article titled “Printed Circuit Techniques”, where they discuss creating circuits on flexible insulating materials such as paper [14]. In the 1950s, N.H. Nashua discussed the printing and etching of flat conductors on flexible materials [15]. The advancement in conductive polymers, organic semiconductors, and amorphous silicon takes a massive pace toward flexibility and processability for applications that require bending, rolling, folding, and stretching, among other properties that were not possible by rigid conventional electronics [16,17,18]. Products with flexible electronics have become a part of everyday life, because of the following advantages: (a) affordable (with the continuous growth in manufacturing and material processing, making flex circuits is affordable now), (b) flexible (cheap substrates such as plastic can be used), (c) customizable (e.g., one can combine systems, sensors and organic light-emitting diodes (OLEDs) displays that trail and interconnect data to users for specific applications, say, health information by monitoring body conditions), (d) innovative (to cover up new applications, innovative designs are realizable, e.g., flexible display), I portable (flex circuits, uses organic thin-film transistors that help products be significantly thinner and lighter and therefore portable). Some common applications are in displays, the automotive industry, biometrics, smart technology (e.g., smartphones, smartwatches, artificial e-skin, smart fridges, smart speakers), and so forth. Some of the areas of flex circuits that are recolonizing the world include industries in auto-manufacturing, medical, energy, bioelectronics, photovoltaics, communication, consumer electronics, architecture, and textiles, extending the functionality of robots and unmanned aircraft through lightweight and conformable energy-harvesting devices and sensors, etc., and have a major role to play for the upliftment of flex circuits innovation [6,9].

Structurally, flex circuits can be single-sided, sculptured, double-sided, multilayer, rigid, polymer thick film, etc., and each structure has its pros and cons and is used for specific applications [19,20]. Since its inception, the multifunctional flexible electron has thriving development in terms of material, structural, and functional designs to fulfill the growing demands on the more advanced functionality of flexible electronics, and of course, there have always been challenges. Some designs even originated from nature and are biodegradable, which may reduce giant electronic waste globally and environmental issues. Additionally, polymers have the advantage of biocompatibility and biodegradability that do not root any adverse effect on the human body and can be smashed into smaller constituent pieces after use. Mostly, a flexible circuit has mechanical and electrical parts. Wiring of interconnects, energy density, and integration now play a vital role in addition to normal functionalities such as excellent mechanical strength, deformability, signal processing, etc. One of the appealing applications of the flex circuit is the development of flexible liquid-gated transistors, recently. Flexible interconnects and circuits based on liquid-gated transistors allow for the creation of transistors that can be bent, stretched, and otherwise deformed without losing their functionality. Thus, flexible interconnects and circuits can be used to create high-performance wearable devices that record heart rate and other vital signs in medical applications [21,22].

Therefore, to have a breakthrough in the field, importance should be given to understanding the physics of the limits of conventional materials and methods and the need to explore new materials and sustainable, reliable, and cost-effective fabrication methods. For example, high adhesion strength and water resilience of flex substrates/systems are some of the key requirements for wearable and skin-attachable electronics, in addition to other requirements, such as energy storage, comfortability, flexibility, stretchability, compressibility, user friendly, etc. Bending and rolling large-area photovoltaics is another exciting application. Assemble of functionalities such as self-powering, energy storage, sensing, mechanical deformability, etc., are often complicated in human-interactive devices, soft robots, medical devices, etc., in complex and dynamic surfaces and/or irregular shapes. Structural design has to be updated or new realistic designs have to be opted for such applications with a hybrid or standalone solution [23,24]. To enhance the detection of health indicators, wearable sensor systems must be able to interface with human skin more effectively. Wearable electronics have significantly improved the integration of Internet of Things (IoT) and Internet of Everything (IoE) applications, with notable advancements in wireless technologies and low-power electronics, particularly for digital health monitoring purposes [25].

**Figure 1:**
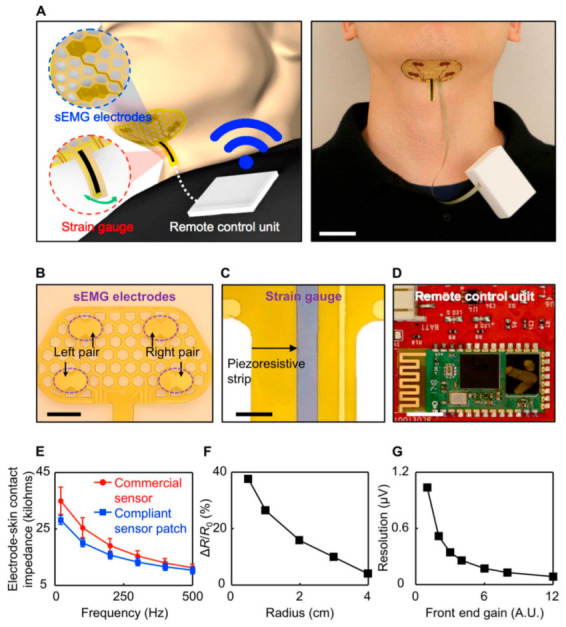
(**A**) The diagram presents a sensor patch that connects to a portable unit clipped onto the wearer’s clothing for remote data transmission and power, as shown on the left side. The right side features a photograph of the same setup, scale bar, 2.5 cm. (**B**) The detailed photograph of sEMG electrodes with a scale bar of 1 cm, while (**C**) features an enlarged photo of the piezo-resistive strip in the strain gauge with a scale bar of 3 mm. An unpackaged integrated circuit chip of the portable unit is displayed in (**D**), with a scale bar of 5 mI. (**E**) The electrode-skin contact impedances of the compliant recording electrodes (blue plot) compared to commercial recording electrodes (red plot). (**F**) Characterization of the strain gauge at various bending radii. (**G**) Characterization of the remote data transmission unit’s resolution at various gain settings. Arbitrary units are represented as A.U. Reproduced with permission from [28]. Copyright 2019, American Association for the Advancement of Science.

The flexible electronics area itself is highly interdisciplinary. It is mostly cross-fertilization between the areas of materials science, electronics and electrical engineering, biomedical engineering, chemistry, physics, computing science, and energy research communities. Novel intrinsically soft substrate materials and structures with durability and conformability, responsive and active devices based on organic and/or inorganic, stretchable electrodes, large-area compatibility, interconnects and circuits, device–bio communicative interfaces, and system-level integration, and innovative process technology are the key areas to be addressed for further strengthening the field [26]. The profound cross-integrate of flex circuits with today’s digital inevitable components based on artificial intelligence, material science, Internet of Things, space science, health science, energy science, and data science, open up a new dimension of applicability and have enough potential to be a technology of everyday life [27]. The sensor patch shown in Figure 1 is a non-invasive, skin-mountable device that monitors muscle activity and laryngeal movement during swallowing tasks and maneuvers. It is specifically designed to fit accurately on the submental area (under the chin) and is characterized by its ease of use, accessibility, reusability, and cost-effectiveness. The patch has undergone preliminary validation and has been tested on a patient with Parkinson’s disease and dysphagia, as well as a healthy control participant. These tests demonstrate the efficacy of the sensor patch for recording submental muscle activity during swallowing [28].

There are two main approaches to achieve exceptional performance in flexible electronics: (1) using soft materials such as molecular and nanostructured materials, polymers, metal foils, and inorganic nanomaterials such as metal nanowires, graphene, and carbon nanotubes as building blocks, due to their unique mechanical, electrical, and optical properties [16]; and (2) employing rational structural design, including island-bridge, textiles, and cracks, to enable rigid materials to bend and deform without breaking [29,30]. Another very important parameter needed in flexible electronics is repeatability. Additionally, softer semiconductors realizing the desired level of stretchability, e.g., polymers and organic transistors, do not have good charge transport properties, and there should be more efforts to increase the mobility in such materials or choose the right material with better transport efficacy [31,32]. To conclude, the important features for the critical advancement of flexible and stretchable devices may be (a) mechanically durable materials, (b) novel processing technologies, (c) electronics and optoelectronics applications, (d) energy storage and generation devices, and (e) biomedical monitoring, etc. [33,34].

## 3. Flexible Interconnects

In this section, we would like to discuss a variety of materials for the application of flexible interconnects. Before starting the discussion, fundamental conditions for flexible interconnects should be addressed. The most critical condition for flexible interconnects is their mechanical properties. Among a variety of mechanical properties, flexibility is the most important one. The flexibility of materials mostly depends on their thickness and physical shape, in addition to the mechanical properties of the material. Typically, thicker materials are stiffer. In the view of material shape, wavy or curvy materials are more flexible than exactly flat materials due to the degree of the freedom of three-dimensional bending at any axis. One of the intrinsic properties of materials that determines their flexibility or stretchability is brittleness. According to environmental conditions such as drying, degree of plasticizing, and so on, the brittleness of materials can vary [35]. Typically, polymers are not as brittle as metal and ceramic at room temperature. With an increase in temperature, the ductility of polymers is increased. Amorphous polymers whose structures are not crystalline behave like glass at a low temperature. However, they may behave as a viscous liquid at high temperatures in which the temperature is above the glass transition temperature (T_g_). Therefore, in order for conducting polymers to be utilized in flexible or stretchable interconnects, conducting polymers with T_g_ around room temperature is essential. When certain flexible or stretchable interconnects are bent by an external force, Poisson’s ratio of the interconnects should be considered. Poisson’s ratio is a dimensionless constant in engineering analysis for determining the stress and deflection properties of materials [35]. For instance, uniaxial stretching of interconnects results in tension along the stretching direction. In contrast, the perpendicular direction of the stretching is subject to compressive stress. Thus, Poisson’s ratio of the materials affects mechanical properties as well as electrical instabilities.

Needless to say, the widely utilized materials in interconnects are metal and metal oxide derivatives. Au, Ag, Cu, Al, indium tin oxide (ITO), and so on, are the representative interconnects in electronics. However, materials based on metal and metal oxide do not have excellent ductility, which means that they are easily fragile and brittle with a certain bending or stretching. In order to overcome this issue, nano-scale structuring can be applied to the metals and metal oxides. The dimensional reduction of materials in a certain direction increases the aspect ratio, which enhances the flexibility. Among a variety of metal nanowires, Ag nanowires are widely utilized in flexible interconnects. Ag nanowires display high electrical conductivity as well as excellent optical transparency. Thus, they can be used in transparent flexible electronics. For example, the deposition of Ag nanowires solution on a target substrate to make a transparent conductive film should percolation networks. Due to the one-dimensional structure of nanowires, the individual nanowires connect together to make a path for charge carrier transport [36]. Other than Ag nanowires, various metal and metal oxide nanowires can be applied in flexible interconnects.

**Figure 2:**
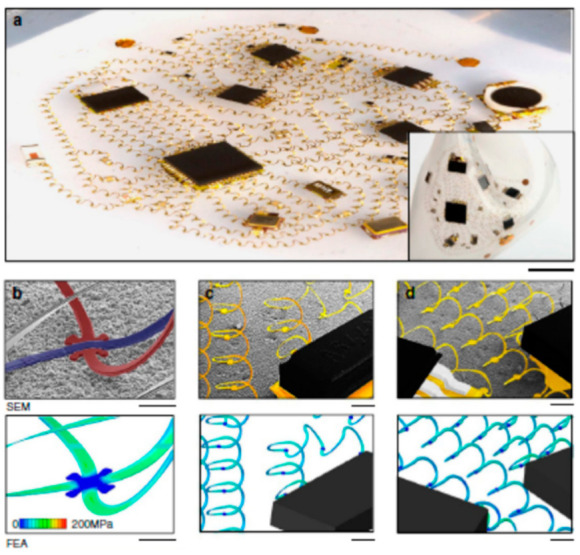
A 3D network of helical coils serves as electrical interconnects for soft electronics. (**a**) An optical image of the system stretched by 50% in two directions. (**b**–**d**) Scanning electron micrographs (top) and corresponding Finite Element Analysis (FEA) results (bottom) of representative regions of the 3D network at electrically isolated crossing points and interfaces with chip components. Reproduced with permission from [47]. Copyright 2017, Springer Nature.

Traditionally most commonly used copper wires have high ductility and malleability and are an excellent conductor of heat and electricity. Of course, copper costs more than fiber optic cable, and its susceptibility to corrosion, shock hazards, etc., are a few drawbacks. Because of the higher conductivity of copper over aluminum and its higher tensile strength (roughly 40% higher), copper wiring is preferred over aluminum [37]. Copper wire [38] or elastic polymeric wires coated with a highly conductive material such as AgNW [39] are used as flexible interconnects. ‘Wavy’ or serpentine shapes have better scalability and stretchability as compared to helical coils and threads and are used as arrays of such applications that will be explained in this section [4,40,41,42,43,44,45]. Honeycomb lattice architecture has air pores to the substrate that may lead to lesser irritation and are comfortable to use [25,46]. Figure 2 depicts a compound-eye architecture that is inspired by the natural spiderweb structure. This architecture shows tremendous potential as a future model for optoelectronic sensing devices that require a wide field of view and high sensitivity to motion. The architecture integrates photodetector pixels with curved microlenses, which are inspired by the fractal web design, into a hemispherical photodetector array. The array incorporates an organic-dye-sensitized graphene hybrid composite that has been reported to be an effective photoactive component with enhanced light-absorbing capabilities. The device exhibits excellent mechanical robustness and electrical properties [25]. This is a good example of the integration of nature-inspired structures for an electronic system with mechanical and electrical robustness.

### 3.1. Fabrications

In flexible and e-textile interconnects, metals (such as gold) are frequently employed as wires or as coating materials on polymeric fibers on flexible substrates (such as glass or PDMS), the fabrication methods for these processes are documented in the literature. In contrast to polymers, metals are stiffer, and even pre-strain gold metallization can buckle after the release of stress, and there is a change in electrical conductivity with stress. More strain can be retained by wavy or serpentine structures. Jang et al. demonstrated an open-mesh, 3D interconnect network of helical microcoils formed by compressive buckling that offers very low modulus, elastic mechanics, which they claim overcomes issues from the 2D network of interconnects [47].

As a result of their excellent mechanical flexibility, superior carrier mobility, and high mechanical stability, carbon nanotubes (CNTs) are promising candidates for high-performance flexible electronics [48]. From a form device point of view, high purity and good homogeneity of the CNT are still a challenge. However, printed devices have substandard electrical performance and uniformity when compared to microfabrication processes, despite printing technology’s promise in large-scale and low-cost manufacturing. One can create a stretchable circuit even when ceramic dielectric layer materials and metal connector materials are still not flexible. For example, Takahashi et al. presented a conformal pressure sensor array with a stretchable single-wall CNT active-matrix backplane [49]. Here, the backplane is made stretchy by laser cutting the substrate into a honeycomb mesh structure and carefully positioning the transistors in the edges where the strain is minimal. Similar techniques have been used by other research groups to have large arrays of sensors. Liu et al. established a multiwall CNT sheet coiled as covering layers on a highly stretched rubbery fiber to fabricate a series of multilayer fibers that could be stretched 1000% for numerous cycles with negligible conductivity change [50].

**Figure 3:**
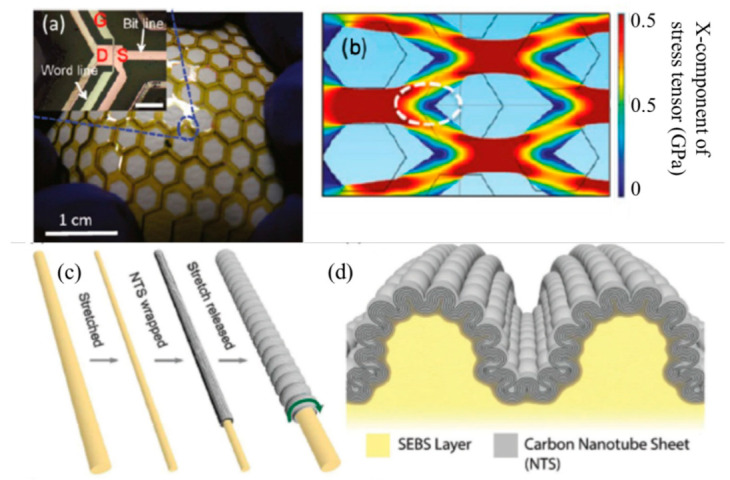
The honeycomb mesh in the stretchable carbon nanotube thin-film transistor (CNT-TFT) backplane architecture is depicted in (**a**), which shows the backplane conformally covering a baseball, and the inset shows a micrograph of one transistor. (**b**) The mechanical strength of the structures with side lengths of 1.25 mm. (**c**) The manufacturing procedure for the core-sheath fiber, with an arrow indicating the direction of the belt. (**d**) A diagram depicting the longitudinal section of the fiber, demonstrating the hierarchical buckling phenomenon. Reproduced with permission from [49]. Copyright 2011, American Chemical Society.

Transparent microelectrodes have these days emerged as a promising method for crosstalk-free multifunctional electrical and optical bio-interfacing. Chen et al. demonstrated a silver nanowire (Ag NW)-based solution-processed and photolithography-based technique that is compatible with potential large-area fabrication transparent microelectrode arrays and interconnects that show high optical transparency, larger mechanical stability, and stabilized electrochemical impedance [51]. Ag NW microelectrodes permit hi-fi co-localized observance of heart rhythms throughout optogenetic pacing and optical mapping. The authors claim that Ag NW transparent microelectrode arrays and interconnects have distinguished applications for multifunctional high-resolution bio-interfaces to quantity and moderate bioelectric organ systems. Figure 3a shows the schematic of the fabrication process, details of which are available in ref. [51].

Although relatively difficult, high-resolution multi-metal layer patterning without lithography has the potential to advance flexible electronics. Li et al. developed a process for producing high-performance OTFT arrays and 3D interconnects for flexible electronics using surface-activation-localized electroless plating (SALEP) [52]. The SALEP method, according to the author, is a low-cost, easy-to-use process that is compatible with large-scale substrate production of single/multi-metal patterns and offers ultra-high resolution, high efficiency, scalability, high conductivity, environmental reliability, mechanical flexibility, and 3D conformations. These features make it a promising technology for the advancement of flexible electronics. The steps to fabricate discrete OTFT arrays through screen printing are shown in Figure 3b. Figure 3c shows the gate deposition on oxide. The patterns are quite strong in terms of electrical reliability, mechanical flexibility, and bending fatigue, as shown in Figure 3d [52].

**Figure 4:**
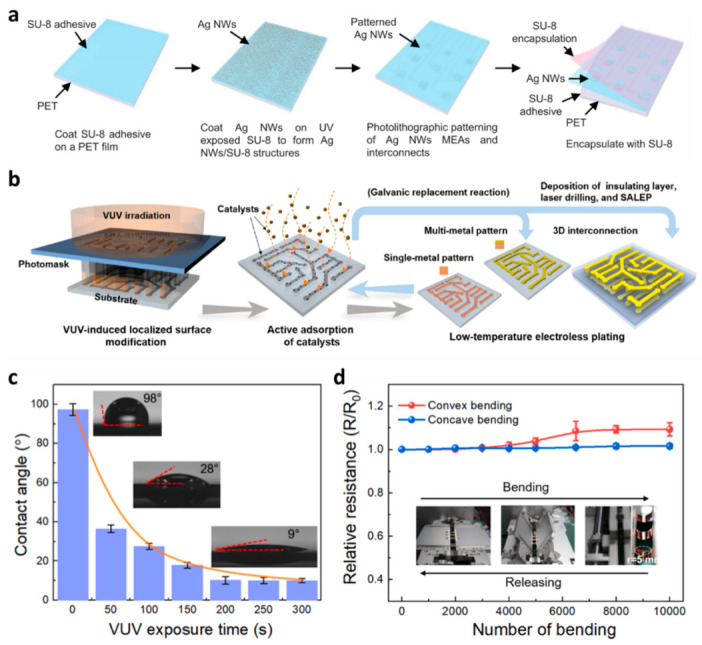
(**a**) A diagram outlining the process for creating flexible and transparent Ag NWs MEAs and interconnects. (**b**) A diagram illustrating the SALEP method for producing high-resolution flexible single and multiple metal patterns [53] (reproduced from John Wiley and Sons with permission). (**c**) The surface contact angle of the COP substrate evolves over time during VUV treatment using the SALEP approach. (**d**) R/R_0_ for Cu/Ni/Au patterns changes based on the number of bending cycles at a 5 mm radius [54] (reproduced with permission from Elsevier B.V. with permission).

Printed circuit boards (PCBs) might malfunction due to copper traces, and the conductive connections are fixed using printing materials such as metal nanoparticles and conductive polymers. Due to its superior electrical and mechanical qualities, graphene is utilized to directly write conductive traces in flexible PCBs, which lack fabrication ruggedness. According to Lim et al., a femtosecond laser direct writing (FsLDW) platform was used to laser-print conductive traces of reduced graphene oxide (rGO) on flexible PCB substrates in order to repair graphene-based electric circuitry [53]. The laser beam for patterning was directed onto the printing area by a pair of angle-scanning Galvano mirrors, as shown in Figure 4a. The rGO was also suitable for flexible electrical traces in terms of excellent resistance performance at different bending angles, as seen in Figure 4b.

Nam et al. reported a method for synthesizing a large-scale, well-dispersed, and high-concentration CuOx nanoparticle (NP) ink by spin coating, which cannot be achieved using commercial nanoparticles [54]. Laser digital patterning at room temperature was used to create finely patterned Cu electrodes with high resistance and mechanical robustness on a variety of polymer substrates. This technology is used to create transparent touchscreen panels and may one day be used to create printable and flexible optoelectronic devices. The schematic of experimental procedures for fabricating Cu electrodes is presented in Figure 4c. Figure 4d shows the laser digital patterning process (LDP) process creating the Cu electrode patterns using the reductive sintering phenomenon of CuOx, details of which are available in [54].

In another study, Ho et al. developed a method to fabricate a mechanically robust and flexible Ni/NiOx-based breath sensor using a one-step digital laser prototyping process of a solution-cured NiOx thin film deposited with NiOx nanoparticle ink [55]. The sensor displays high sensitivity with rapid response and recovery times (1.4 s/1.7 s) at low operating temperatures (50 °C) and exhibits strong electromechanical stability during flexural conditions and cyclic bending tests. Figure 4c,d depict a schematic and microscopic image of the fabricated sensor and its corresponding responses, and the detailed methodology is available in [55].

**Figure 5:**
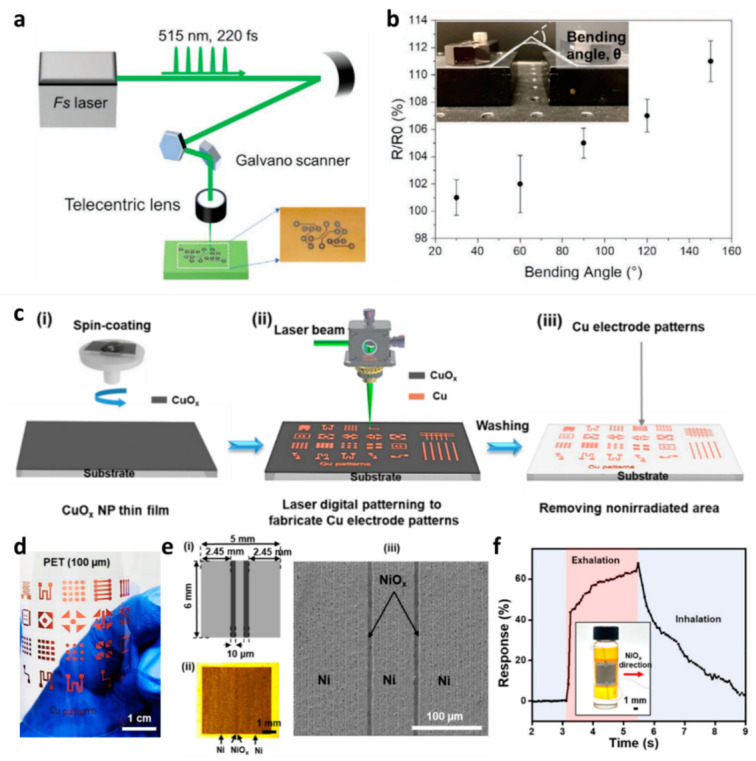
(**a**) Schematic of the FsLDW system used for printing the rGO interconnects. (**b**) Change in resistance due to bending angle. Inset shows the photograph of the measurement system [56] (reproduced from Elsevier B.V. with permission). (**c**) Schematic of experimental procedures for fabricating Cu electrodes. (**i**) CuOx thin films formed on various substrates by spin coating. (**ii**) Laser digital patterning process for producing Cu electrodes. (**iii**) Washing process to remove the nonirradiated areas. (**d**) Arbitrary Cu electrode patterns generated using the laser digital patterning (LDP) process for PET (thickness: 100 μm). (**e**) (**i**) Schematic drawing, (**ii**) optical microscopy image, and (**iii**) scanning electron microscopy (SEM) image of the Ni/NiOx-based breath sensor. (**f**) Response curve of the Ni/NiOx-based breath sensor to the normal breathing rate at the operating temperature of 50 °C under a bending condition [57] (reproduced from IOP Publishing, Ltd. with permission).

A contact printing-based method, based on an adhesive force between interfaces, for fabricating a liquid metal (LM) pattern on a PDMS substrate was reported by Kim et al. [56] Contact printing is a fabrication process in which LM film is transferred to another mold, and the authors claim that this has application to wearable self-powered electronics. The electrical conductivity of the patterned LM was sufficiently high to be used as an electrode and was semitransparent, flexible, and stretchable for next-generation wearable electronics. Figure 5a clearly shows the steps for LM transfer to the PDMS mold, and the image of the fabricated LM electrode is shown in Figure 5b.

Park et al. demonstrated a system scheme for fabricating stretchable active matrices of oxide thin-film transistors (TFTs). The place TFTs and go points of two metallic layers on stiff islands are monolithically integrated with gallium-based liquid steel interconnects within an elastomeric matrix [57]. The process has high integration density and mechanical durability, which provides stable operation up to 40% of stretching for a 4 × 4 matrix, as shown in Figure 5c.

### 3.2. Interconnect Types

Gold (Au) is often treated as an excellent conductor and interconnect material because of its fine conductivity and stretchability (until 20%) [58]. However, due to resistance change under stress, it cannot be used as an intrinsically stretchable conductor/interconnect [59,60]. Gold nanowires and conductive percolation networks with nanowires, exhibit excellent conductivity and stretchability and have the norms for flexible interconnect; however, there is an issue with the point contact at NW–NW junctions as aforementioned. Silver nanowire networks exhibit optical, mechanical, and electrical properties [61,62]. There are many reported works on copper nanowire networks that show their applicability as flexible interconnects. Stretchable metallic NW conductor networks are also embedded into a polymer matrix that has minimum resistance change under strain. The issue with metallic NW-based networks is the weak junction contacts, which reduce mechanical and electrical performance. There are many reports for improvements in contact at nanowire junctions, and the details are elaborated by Zhang et al. [2].

Among low-melting metals, Ga-based alloys that have a lower vapor pressure at room temperature exhibit intrinsically stretchable conductors and are less toxic than liquid metal, e.g., mercury (Hg) [63]. Of course, liquid metals are compressed into elastomers that prevail stretchability for use in electronic devices. Thin-film transistor-based liquid metal interconnects have almost nil resistance change until 50% strain [64]. Liquid metals have some fundamental shortcomings compared with metals such as toxicity, low conductivity, and low manufacturability, which sets drawbacks for their application in flexible interconnects in the near future [65]. Carbon nanotubes (CNTs) and graphene are extensively studied for flexible conductors and interconnects due to their excellent electrical and mechanical characteristics, and they are lightweight. However, due to the fabrication challenge, the use of these interconnects is still a challenge [5,25].

Other than metals, conducting polymers are one of the widely used flexible interconnects. Conducting polymers contain high mechanical flexibility and tunable conductivity depending on the chemical composition. The fabrication of flexible interconnects using conducting polymers is readily accessible via either a solution- or a vapor-way. Due to their merits in low-cost fabrication, mass production of the conducting polymers is realized. However, conducting polymers have become intrinsically unstable over time owing to a spontaneous degradation by ambient conditions such as light, temperature, oxygen, and water molecules in the air. To solve this environmental instability of conducting polymers, active research in chemical and mechanical approaches has been carried out for a long time. Polyaniline, poly (3,4-ethylenedioxythiophene): poly (styrenesulfonate) (PEDOT:PSS), polyacrylonitrile, and so on are representatives of conducting polymers. Among them, PEDOT:PSS is commercially available [66,67,68]. The PEDOT:PSS is highly flexible and stretchable as well as optically transparent, which means it is applied in various electronic fields such as organic photovoltaic devices, organic field-effect transistors, and organic sensors. The conductivity of PEDOT: PSS ranges from 10^–4^ to 10^3^ S cm^–1^ according to synthetic routes, doping agents, and types of pre/post−treatment conditions.

**Figure 6:**
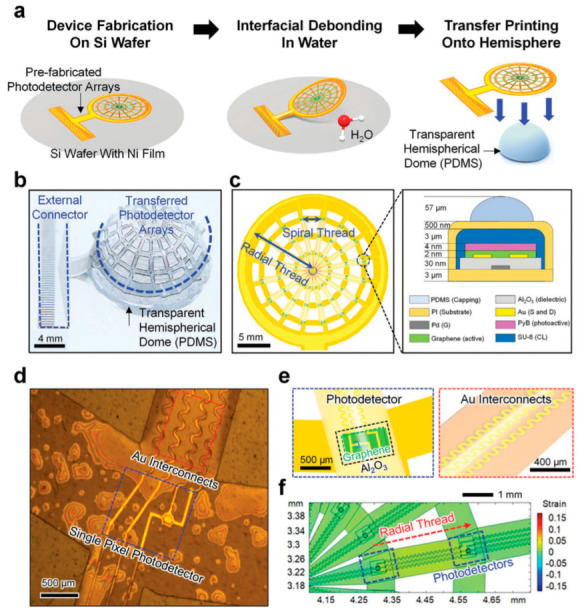
The fabrication method and device layout. (**a**) A schematic depicts the process of assembling the hemispherical photodetector array. (**b**) A photograph shows the device placed on a 15 mm diameter transparent hemispherical dome made of PDMS. (**c**) A schematic illustrates the fractal web design’s structural layout, with an inset showing the cross-sectional view of a single-pixel photodetector. (**d**) A microscopy image displays the single-pixel photodIctor. (**e**) Enlarged schematic illustrations depict the single-pixel photodetector (**left**) and the Au interconnects’ serpentine traces (**right**). (**f**) FEA results display the stretching behavior of a single radial thread. Reproduced with permission [25]. Copyright 2020, John Wiley and Sons.

Intrinsically stretchable conductive polymers (e.g., polyaniline (PANi), polypyrrole (PPy), polythiophene (PTh), poly (3,4-ethylene dioxythiophene) (PEDOT), and their derivatives) are found to have better stretchability than metals and carbon-based nanowires. However, its main drawback is its simultaneous maintenance of both mechanical and electrical performance [69]. A polymer-based cross-linked structure with good stretchability, low Young’s modulus, and outstanding fatigue resistance are reported [70]. Conductive composites (CC) and CC filled with metallic nanoparticles and carbon materials are also studied by many researchers for the possible use of conductors and interconnects. By implanting conductive fillers in a polymer matrix, the composites are enabled to exhibit simultaneous improvement of conductivity and stretchability. Conductive composites are now used in practical applications such as wearable devices, multifunctional sensors, energy harvesters, and biomedical fields [2]. Figure 6a–c show the assembly process of a hemispherical photodetector array, using a Si wafer with Ni (100 nm thick) and polyimide (3 μm thick) layers for separation and support. The photodetector array was made by sequentially depositing and patterning an epoxy-based negative photoresist, a Pyronin B (PyB)-doped graphene sheet, a dielectric layer (Al_2_O_3_), and metal electrodes (Pd and Au) on top of the PI layer. Figure 6d,e exhibit a microscopy image and an enlarged schematic of the single-pixel photodetector and the serpentine traces of Au interconnects positioned along radial threads beneath the ≈57 µm thick PDMS capping layer. Figure 6f displays the PDMS capping layer’s stretching behavior with strains of <4%.

### 3.3. Interconnects in E-Textiles

Flexible electrical systems incorporated in textiles offer a multifunctional electronic platform to yield flexible, conformable, and large-area textile-based electronic systems (larger than silicon wafers) and have applications in wearable electronics, healthcare, communication, and entertainment, sports, space, security and surveillance, etc. [7]. There are many research papers and review papers on flexible interconnects for flexible textiles. One of the important parameters here is the fiber-to-fiber interconnects within textiles with good conductivity under strain for use on the non-planar surface of the body [71]. Silicon-based technologies have not succeeded in e-textile to achieve application demand because of mechanical incompatibility, which can be achieved by a polymer-based fibrous substrate with exceptional properties [72]. Interconnects that connect flexible substrates and electronics are a challenge [7]. Ultra-thin monocrystalline silicon integrated circuits (ICs) fabricated on flexible foils can deliver great electrical performances while withstanding mechanical flexibility with minimum power dissipation. The dynamic bending reliability (fatigue) of such ICs is quite important. Palavesam et al. found that gold has better fatigue performance compared to copper and aluminum [73].

In addition to the flex circuit being portable, flexible, small or robust, etc., interconnects used in flexible circuits should be of high quality in terms of better conductivity under stress, efficient signal transmission with a minimum loss, bendability, ability to roll, and compress, good adhesion, low cost, high density, and fabrication and characterization compatibility, that consumes lesser power and most importantly compatible with the target host body comfortably, etc. In addition, materials that enable self-healing are lightweight, biocompatible, have anti-oxidative properties, transparency, etc., and have added advantages. Broadly, two different practices are followed for the mechanical stretchability of interconnects, namely, (a) using inherently stretchable conductive materials and (b) shape engineering of non-stretchable conductive materials into wavy, meandering, or pre-stretched structures to remove the tensile strain under stress. Stretchability is an intrinsic property that cannot be attained by the structural design of patterning. Stretchable conductors regulate the performance of integrated devices and systems. Often, in wavy or meandering structures, stretchability and electrode density make a tradeoff; pre-strain structures have direction-specific stretchability. It is preferable and often a market demand to have the fabrication process as simple as possible to reduce production costs. Intrinsically stretchable materials do not need additional process steps and therefore offer reduced cost. Lesser space is required, and they are a better fit if the desired stretchability can be achieved for an application [2,74,75,76,77]. Conductivity depends on the dimension of the interconnects as well as the material’s properties. A structure/design may face continual static or dynamic strains in multiaxial directions; therefore, the flexible material must have excellent mechanical properties. To achieve innovation in flexible interconnects, one must work towards materials, mechanical, electrical, environmental (biodegradable), and under harsh environments (underexposure to light, temperature, humidity, etc.) as well as reliability features of flexible interconnects. The interconnects are expected to have excellent frequency response, i.e., bandwidth. The bandwidth of a transmission line determines its signal-carrying capacity at different frequencies. A higher bandwidth indicates that more signals of different frequencies can be transmitted via the interconnects.

Metallic nanomaterials, liquid metals, carbon-based nanomaterials, conducting polymers, and conductive composites are some of the intrinsically stretchable and conductive materials [78]. One-dimensional (1D) metallic NWs and nanotroughs improve the stretchability of metallic blocks. Of course, weak contact junctions sink their mechanical strength and conductivity. Conductive percolation networks formed by metal nanowires have both excellent conductivity and stretchability [59]. Liquid metals have restrictions due to their toxicity, low conductivity, and manufacturability disadvantages [2]. Carbon nanomaterials form a good contact [79]. Conducting polymers have better stretchability compared with metallic and carbon nanomaterials; however, as expected, they have inferior conductivity. Embedding conductive fillers in a polymer matrix, conductive composites demonstrate both conductivity and stretchability [80].

The resistance of the conducting wire needs to be minimum. The distance between two conducting lines must be optimized to minimize electromagnetic coupling, which may lead to cross-talk due to voltage change across the interconnect lines. Fundamentally, metals and metallic nanowires, liquid metals, carbon nanomaterials, coated materials, conductive polymers, and conducting polymer composites are used as interconnects depending on applications [81]. For example, a stretchable electrode should have negligible resistance change under strain; on the other hand, a large change in resistance with a slight tensile strain is essential for mechanical sensors. It means that the selection of stretchable conductors should be based on practical applications [2].

Silicon interposer-based 2.5D integration delivers a high-bandwidth and low-energy interconnect platform for heterogeneous systems [82]. Yang et al. reported an interconnect technology platform to enable a large-scale‘ interposer tile’ and ‘silicon bridge’ [83]. The common thick-film methods have inferior resolution below 50 Jlm. Direct-write methods are suitable for lower scale (i.e., from 1 Jlm to 100 Jlm) ranges at the cost of low throughput and high production cost. Bhattacharya et al. proposed a modified mill and fill (M&F) interconnect technology for high-resolution, high-throughput, and high-density conductive trace patterns on flexible substrates [84]. Different topologies are used as interconnect structures such as serpentine, buckled geometry, honeycomb structure, etc. Each structure has its own merits and demerits in terms of electrical and mechanical performance. Arafat et al., demonstrated a cost-effective fabrication process of stretchable Indium interconnects on an elastomer PDMS substrate with engineered interfaces to increase routing density and has a stretchability of 35% [85]. In conclusion, the important challenges in textile interconnects are (a) materials and (b) routing signals/power and their connections with terminals of rigid circuit components.

### 3.4. Integration of Interconnects

Integration of all electronic components and ICs on the flexible substrate with optimum space and power is very important for sustained performance. Soldering and welding are very old and common methods for connecting two wires; however, these processes generate heat, stress, brittleness, etc. In addition, corrosion developed in soldering is not biocompatible. Mechanical gripping by crimping or stapling flexible conductive interconnects to other circuit elements is more reliable in terms of flexibility [86]. Conductive adhesives are another choice here; however, they offer different electrical properties in different orientations [87]. Polymer composites work as better adhesive material at the cost of lesser electrical conductivity, thermomechanical fatigue, and impact strength [88,89]. For e-textile, inkjet printing is commonly used to create conductive patterns by using conductive inks [81]. In summary, there is still much to be achieved in integrating different circuit elements for wearable e-textiles. The main challenge is efficiently routing electronic circuits on textile substrates using currently available rigid integrated circuits and components with suitable interconnects. A hybrid solution that combines current rigid semiconductor/metal/dielectric components with a soft and flexible fibrous network may offer a better integration strategy, and optimal approaches are being sought.

With all the advantages of flex circuits, there remain a few still unsolved challenges such as matching the performance of the status quo, packaging, and interconnects, and lack of practical integration schemes, proper passivation, or screening techniques of interconnects to cheerfully complement the prevailing state-of-the-art technology. An ultra-high-density array of sensors for healthcare and IoT applications for continuous monitoring and spatiotemporal mapping activities is a challenge in terms of flexibility, integration, interconnects, battery life, etc. However, as the number of the vertical electronic layer increases, extraneous electrical interference between adjacent layers can manipulate electrical signals or degrade the signal-to-noise ratio, eventually causing systematic errors in sensitive analog electrical measurements. It is therefore vital to eliminate all possible sources of interference by proper passivation or screening techniques of interconnects. In terms of flexibility, a standalone electronic system with 3D integration of sensors on one side and readout circuit and data processing units embedded in the polymer and the other side for antenna and energy harvester (photovoltaic), etc., was reported by Shaikh et al., which has a bending radius of 1 mm. The circuit has reliable electrical interconnection and compatibility with existing CMOS technology, in addition to the fact that it is suitable for large-scale manufacturing [90]. The authors also reported a non-invasive ‘Marine-Skin’ platform to monitor deep-ocean monitoring with a flex circuit that has again an integration challenge.

Because the Internet of Everything (IoE) is still in the developing stages in many ways, the adventure of flexibility and stretchability in real-world applications needs proper and optimum integration of interactive system incorporation of related circuits such as data acquisition circuits (e.g., sensors) [91,92], data processing and decision-making circuits (e.g., logic circuits and elements), data storage (e.g., memory devices) [93] communication (radiofrequency and Li-Fi elements) [94], decision accomplishment (actuators) [95], and efficient power management (energy scavenging and energy storage) [96]. For example, in real-time healthcare monitoring applications, electronic devices/circuits might work on a planar/curvilinear surface around the clock internally/externally, which obliges the achievement of the physical flexibility, stretchability, and configurability of electronic devices, in addition to the fact that the system integrates all circuits. In short, in addition to having biological signals processing, continuous health monitoring, higher demand for a larger cache memory with more processing speed (implies increased form factor), and better interconnects are the tradeoffs and design parameters, in addition to the flexibility and stretchability of a flex system. Diminished devices/systems with complex fan-outs are progressively stimulating for automated manufacturing because tiny pins on each side of the integrated circuits (ICs) require greater accuracy and precision, and the same is the case for flex circuits. A parallel assembly used in conventional CMOS technology has challenges in flex circuits [97]. A single-device interconnect failure may lead to failure of the block, which implies an entire system failure. Often in today’s applications, e.g., in healthcare, an array of sensors (sensor matrix) is required, and connecting and accessing individual sensor output are quite challenging for a non-planar surface. In such high-density electrode systems, one of the critical challenges is how efficiently the individual sensor or the channel responds to the signal without being affected/induced by changes in adjacent electrodes (also called spatial resolution), which involves noise cancellation issues and the way how the sensors are isolated [98]. Mapping these individual sensors is another important topic for more common applications such as healthcare, aerodynamic monitoring, etc. With more sensor density, the complexity of managing spatial resolution, mapping, fabrication challenges, interconnection, space and power issues, etc., increases. Shaikh et al. reported a very sensitive and comprehensive CMOS technology-compatible process to fabricate a flexible (bending radius up to <500 μm) high-density pressure sensory (1 million sensors) patch for mapping activities with 15,625 sensors per mm^2^ with a spatial resolution of <10 μm) and electrode size variations of 50 nm to 500 nm. The mapping of an array of millions of sensors is a new opportunity, especially in biomedical applications. To have ever-increasing performance enhancement in semiconductor technology, new transistor structures such as FinFET, silicon nanotube FET, wavy channel thin-film transistors [99], etc., are being used. To effectively attain the advantages of these transistors, fresh innovations in chip architectures are desirable both in CMOS as well as flex circuits.

**Figure 7:**
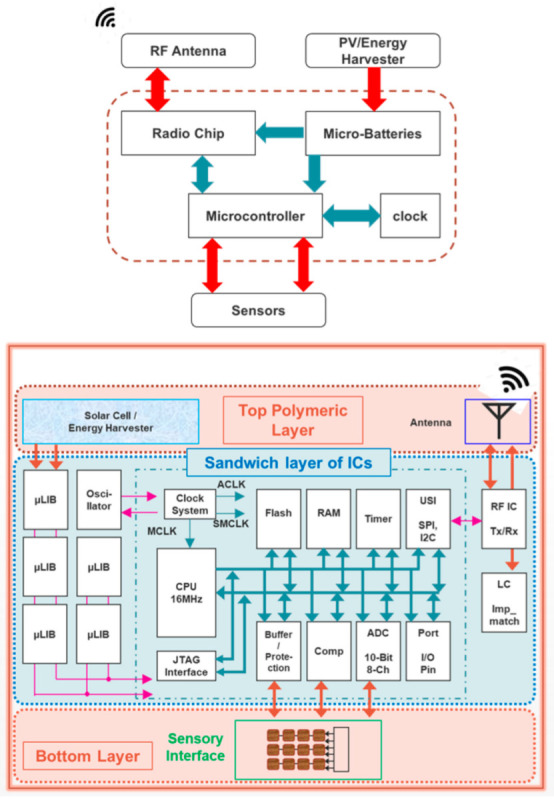
The diagram image shows the arrangement of components for electronic systems that can function independently. It includes a general overview diagram at the top and a more elaborate diagram of a 3D-coin independent system at the bottom. Reproduced with permission [90]. Copyright 2021, Institute of Electrical and Electronics Engineers.

The stacking of several silicon chips together increases the density of the transistors and enables circuit designers to incorporate more functionality into a single chip. The stacked chips can be made with dissimilar channel materials or specific process modules. Shaikh et al. fabricated and characterized a heterogeneous 3D chip to gain a physically flexible standalone CMOS electronic system using a coin architecture by integrating heterogeneous chip elements (called 3D-coin architecture) with smaller interconnects, making the circuits faster (Figure 7). Smaller interconnect wires have many advantages such as lower power dissipation because of Joule heating, smaller parasitic capacitance (which additionally reduces the power consumption), higher bandwidth, vertical interconnect buses, lesser possibility of reverse engineering (secure design), etc. In short, as they report, in a 3D-coin architecture, heterogeneous materials and technologies such as CMOS, polymers, and graphene are assimilated together on a single platform. They used a sequential-etch-back (SEB) technique to transform the CMOS ICs into flexible ICs [100].

## 4. Flexible Batteries

The principal job of a battery is to store energy and power electronic devices. Traditionally, Li-ion batteries are used dominantly to power electronic circuits. However, because of their non-flexible nature, such batteries are difficult to use in flexible circuits. Over recent years, there has been tremendous progress in flexible batteries.

Conventionally in any kind of electronic circuit or system, the primary design criteria are to achieve high-speed and low-power operation. Both of these characteristics are difficult to fulfill simultaneously, and therefore tradeoffs are always looked into as per applications. Flexible electronics are generally designed for low-power usage. Due to the pliable nature of these applications, the use of flexible and stretchable batteries is essential for making the concept of wearable and compliant electronics a reality, especially when used on non-flat surfaces. However, the development of flexible batteries that rely on traditional planar structures, which require the stacking of battery components, will require technological innovation [101,102]. Flexible batteries have been evolving in the last decade from coaxial fiber batteries in 2012 [103] to recent polymer/polymer separator or polar gel polymer electrode methods in 2019 [104,105].

In recent times, there has been a surge in the development of innovative techniques for designing flexible batteries. These approaches encompass a wide range of concepts, including kirigami, origami, bridge-island, arched electrode architecture, winding fibers around elastic support, embedding battery active materials within stretchable fabrics, and embedded nanowire elastic conductors, among many others. These diverse methods demonstrate the ongoing effort to create more versatile and adaptable batteries that can power the next generation of wearable and flexible electronics [40,106,107,108,109,110,111]. Flexible batteries that are concerned with safety and of course cost are of utmost concern. Zamarayeva et al. demonstrated mechanically robust current collector geometries such as serpentines or helical springs to support remaining battery components and have a form factor of a flexible wire (flexing > 17,000 times) [112].

Rechargeable smart energy storage in terms of sustained energy density, power density, cyclability, and technical maturity are some of the parameters that are expected from a smart battery. Lithium-ion (Li-ion) batteries fulfill most of these parameters to some extent. This is in addition that the battery should be bendable, twistable, stretchable, and ultra-thin so that it adjusts with mechanical and electrochemical deformability for use in flex circuits [113]. However, Li-based devices have some weaknesses such as toxicity, flammability, environmental issues, natural abundance limitation, etc. Non-lithium batteries are also studied for safety and low cost. Aluminum and copper foils that are traditionally used as current collector materials for anode and cathode are not a better fit for flexible batteries because of their low mechanical compatibility. Carbonaceous and polymeric materials (CNTs, graphene) have high flexibility, better conductivity, lightweight, and large surface area and are now looked at as alternatives as discussed above. However, in choosing energy-active materials in addition to mechanically sound architecture and configurations for electrochemical performance, one must be careful because of the constraints such as (a) connection of carbon and polymer matrices and (b) reliability of adhesion between active materials and current collectors because of increased hierarchical pores that deteriorate charge transfer and capacity delivery [114]. Another very important component of the flexible battery is the selection of electrolytes (with facile ion transport and minimized self-discharge). Solid-state electrolytes appear to be a practical choice in flexible electronics compared to liquid counterparts for tiny electrolyte leakage, easy cell patterning, mechanical reliability, wide electrochemical window, etc. [115,116]. Separators should be of optimum design and quality for excellent ion transport [8]. To reduce electronic waste, biodegradability concerns are also on the list. In summary, well-performing Li-ion batteries with rigid and brittle features cannot directly be implanted into flexible devices. For a breakthrough in the flexible battery arena, energy-efficient materials in terms of mechanical and electrical performance, battery processing techniques for cost-effective mass production, compatibility with others, compatibility under persistent dynamic mechanical stresses, long-lasting current collectors, electrolyte/separator, packaging have to be redesigned/relooked, and this is a challenge as commercially most successful and matured conventional Li-ion battery does not offer these advantages. Alternative batteries using organic and/or polymer electrolytes, aqueous cells, and 3D nano-micrometer-scale supercapacitors may emerge in the near future, offering improved performance and flexibility for next-generation electronic devices.

Because all components of flexible electronic devices have to go hand in hand, interconnects that consume minimum power and are easy to fabricate are always preferred.

**Figure 8:**
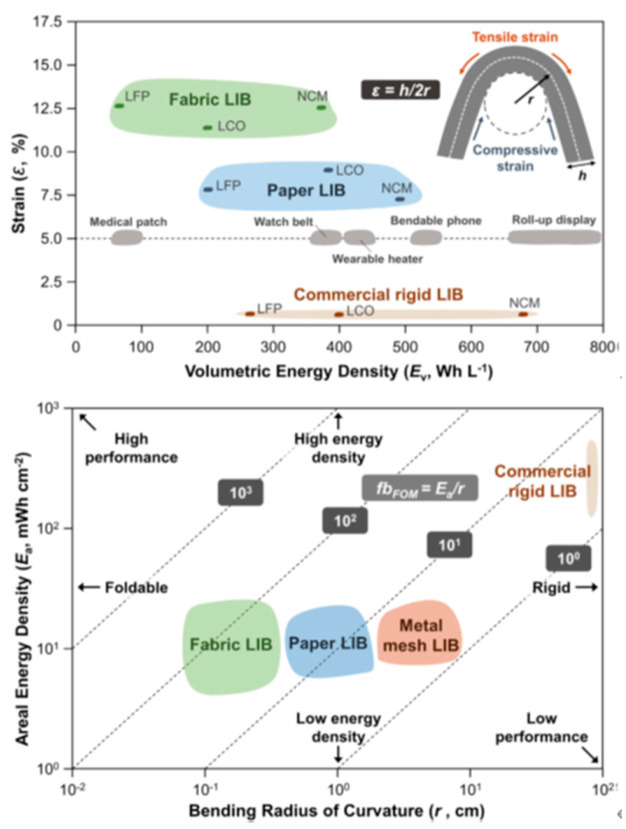
Strain (**e**) with respect to volumetric energy density ($E_{v}$) of flexible battery (upper). Li-ion batteries (LIBs) are grouped according to the use of materials, including metal foil, carbon paper, and conductive fabric. Figure of merit for flexible batteries (down). Reproduced with permission [8]. Copyright 2020, John Wiley and Sons.

Often, the bending radius of curvature (*r*) is used to evaluate the flexibility of flexible batteries, a smaller *r* indicates higher flexibility [117]. Applied strain (∈) on the outer cell depends on both r and cell thickness (h). When strain exceeds its critical value, electrical contact is lost, and mechanical fracture happens to lead to cell failure. In conclusion, flexible batteries for flex circuits are still a developing pitch, and their improvement will improve flexible and wearable electronics applications and reliability. Achieving higher energy density and flexibility are the key challenges. The figure of merit (FOM) for a flexible battery is determined as ($E_{a}/r$), where $E_{a}$ is the areal energy density of the battery and studies, and r is the bending radius of curvature. The bending strain for volumetric energy density is shown in Figure 8, showing the figure of merit of flexible batteries. The maximum bending curvature of flexible batteries can be predicted by strain, while the volumetric energy density of the battery can indicate the suitability of the cell structure for practical applications. The plot highlights the requirements of flexible batteries for various wearable and flexible applications, such as medical patches, wearable heaters, bendable phones, and roll-up displays. To compare flexible batteries composed of different materials, structures, and dimensions, a figure of merit (FOM, $E_{a}/r$) based on the areal energy density versus bending radius of curvature was proposed. A high FOM represents a high-performance flexible battery. The FOM for lithium-ion batteries (LIBs) made with various current collectors is presented. FOM also enables the comparison of the performance of a thin, single-pair-electrode cell with that of a thick tandem cell. The important parameters that define the battery performance are (a) flexibility in terms of bending, foldability, twisting, etc. [8]. Mechanical properties of the materials have a greater role to play here. (b) Energy and power densities [118]. For stretchable systems, the areal and longitudinal energy/power densities are most consistent in addition to gravimetric ($Wh\;{kg}^{-1}\;\&\;W\;{kg}^{-1}$) and volumetric $Wh\;I^{-1}\;\&\;W\;I^{-1}$ energy and power densities Flexible rechargeable lithium-ion batteries: advances and challenges in materials and process technologies [118,119]. Therefore, for innovation in flexible batteries, attention needs to be given to achieving these parameters.

## 5. Summary and Outlook

The progress of interconnection technologies for flexible electronics looks promising, and it is expected that more breakthroughs will be achieved in the near future. The advancement of interconnection technologies is critical for the growth of flexible electronics in various applications. For instance, in the field of wearable electronics, interconnection technologies will play a pivotal role in enabling communication between devices and with other electronic systems, facilitating improved functionality and interaction. Similarly, in the medical sector, interconnection technologies will be vital for developing new medical devices, such as flexible sensors and implantable devices, which will enhance patient monitoring and care. The development of interconnection technologies will also play a crucial role in the advancement of new energy storage and conversion devices, such as flexible solar cells and batteries, by enabling the integration of different components for optimal functioning. However, further research is needed in some areas, such as:

**Material Development**: Developing new interconnection materials with improved reliability, scalability, and cost-effectiveness will exercise a central role in the continued growth of the field. Research into the development of novel interconnection materials, such as graphene and carbon nanotubes, has the potential to greatly improve the performance and reliability of interconnection technologies. They have unique physical and electrical properties that can enhance the connectivity between the various components in a device. Graphene and carbon nanotubes have high electrical conductivity and mechanical strength, which makes them ideal for use as interconnections. In addition, they have low thermal resistance and are flexible, which allows them to withstand repeated bending and folding without breaking or losing their electrical conductivity.

**Fabrication Techniques**: Improving fabrication techniques to enable large-scale production of interconnection structures will be crucial for commercialization and widespread adoption. The development of new fabrication techniques, such as roll-to-roll processing, transfer printing, direct writing, lithography-based techniques, laser printing, and inkjet printing, has the potential to greatly reduce the cost and complexity of interconnection technologies.

**Integration with Flexible Electronics**: Integration of interconnection technologies with flexible electronics will be essential for realizing the full potential of these devices in various applications. Additionally, the integration of interconnection technologies with existing electronic systems, such as mobile devices and wearable technology, will be critical for the continued growth of the field.

**System-level Integration**: Developing interconnection technologies that can integrate flexible electronics with existing electronic systems will be necessary for realizing the full potential of these devices in various applications. Furthermore, the integration of interconnection technologies with other components, such as flexible displays and sensors, may also pose a challenge, particularly in terms of ensuring reliable performance and consistent quality.

**Characterization and Testing**: Characterizing the electrical, mechanical, and thermal properties of interconnection materials and structures will be critical for the continued development of new interconnection technologies. Another important area of research will be the development of new characterization and testing methods for interconnection technologies. This will be necessary for the development of new interconnection materials and fabrication techniques, and the optimization of existing interconnection technologies.

**Sustainability**: As the demand for flexible electronics grows, it is important to consider the environmental impact of these technologies, from the production of the materials to the end-of-life disposal of the devices. To address these concerns, research and development efforts will need to be focused on developing interconnection technologies that are environmentally friendly, from the materials used to the manufacturing processes. For example, research into the use of renewable energy sources in the production of interconnection materials, such as graphene and carbon nanotubes, will be important for reducing the environmental impact of these technologies. Additionally, efforts will need to be made to improve the recyclability and end-of-life disposal of flexible electronics, including interconnection technologies. This will involve developing new methods for recovering valuable materials from end-of-life devices and repurposing them for new applications, as well as developing new recycling technologies that are safe, effective, and cost-efficient.

**Reliability**: The development of reliable flexible electronic systems is essential for the widespread adoption of this technology in a variety of applications. Flexible electronic systems are susceptible to a variety of reliability issues, including mechanical fatigue, environmental degradation, and changes in electrical conductivity. These issues can be caused by several factors, including the materials used to create the devices, the manufacturing process, and the environmental conditions in which the devices are used. In order to quantify the reliability of flexible electronics, electrical properties (conductivity, resistivity, sheet resistance, etc.) would be measured several times under various mechanical stresses (bending, twisting, stretching, etc.). To improve the reliability of flexible electronic systems, both new materials and manufacturing processes that are more resistant to these issues, as well as designing devices with more robust structures, should be developed concurrently.

Overall, the field of interconnection technologies for flexible electronics is rapidly advancing, and there is significant potential for further progress in the near future. Through continuous research and development, flexible electronics are likely to become increasingly essential in our daily lives, transforming the way we live, work, and interact with technology. To sustain this progress, it is critical for researchers, industry leaders, and policymakers to collaborate and invest in research and development initiatives. In summary, the prospects for interconnection technologies for flexible electronics are promising, but it is also essential to consider the environmental consequences of these technologies. By emphasizing sustainability and environmental responsibility, we can ensure that the future of interconnection technologies for flexible electronics is optimistic and has a positive impact on the environment.
